# Supplementary material for: GRIPT: a novel case-control analysis method for Mendelian disease gene discovery
Source: Genome Biol. 2018 Nov 26;19:203. doi: 10.1186/s13059-018-1579-x (PMC6258408; doi:10.1186/s13059-018-1579-x)
Supplement: Supplementary file 2 — Figure S1. Benchmark of GRIPT with CADD, REVEL, and DANN scores on 400 Mendelian disease genes. Figure S2. Test the impact of patient cohort sizes with REVEL and DANN scores. Figure S3. Test the impact of population stratification with REVEL and DANN scores. Figure S4. Test the impact of variant frequency filtering with REVEL and DANN scores. Figure S5. The main procedure of simulation analysis. (PDF 3207 kb) [file 13059_2018_1579_MOESM2_ESM.pdf]

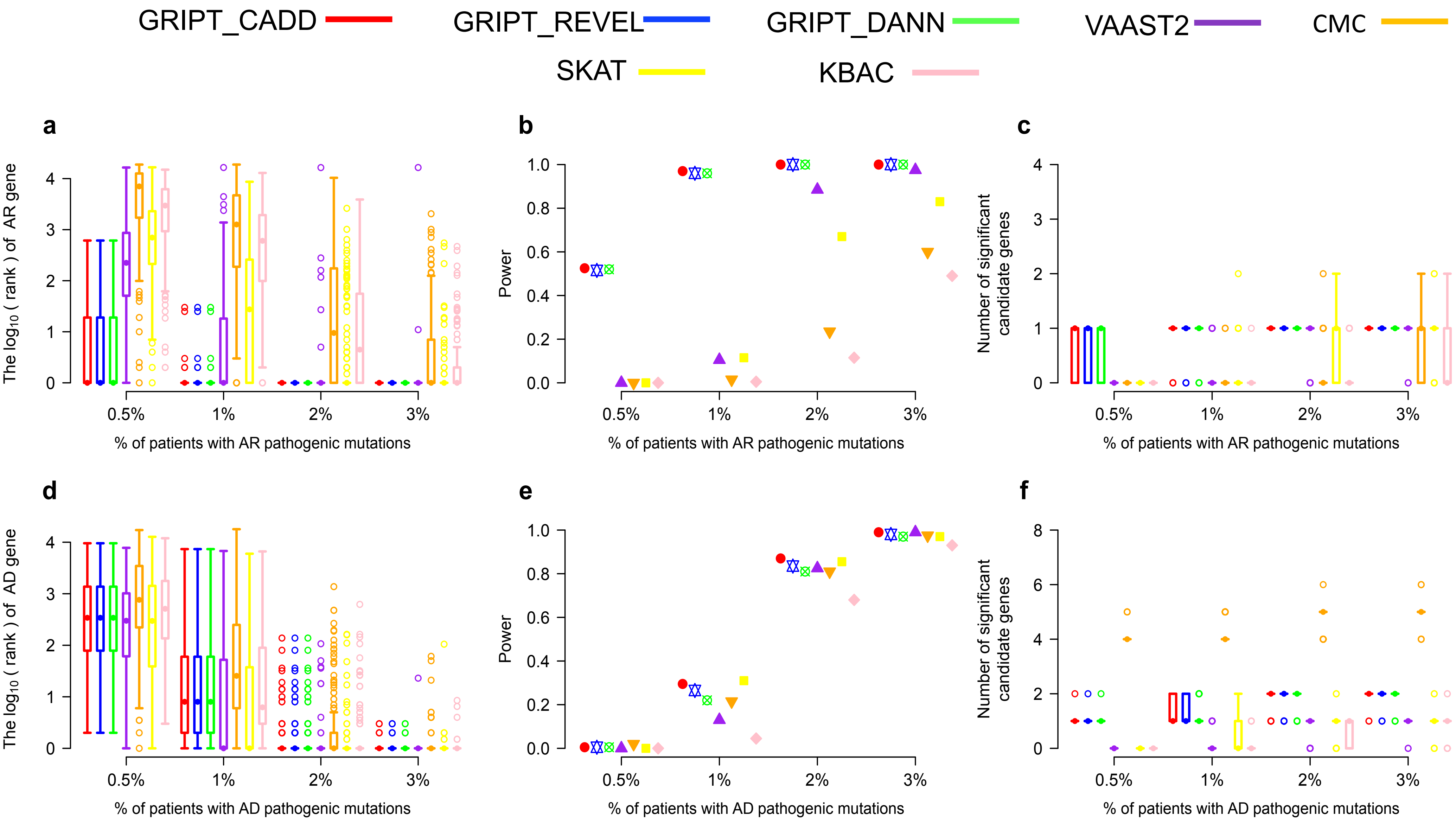

Figure S1. Benchmark of GRIPT with CADD, REVEL and DANN scores on 400 Mendelian disease genes.

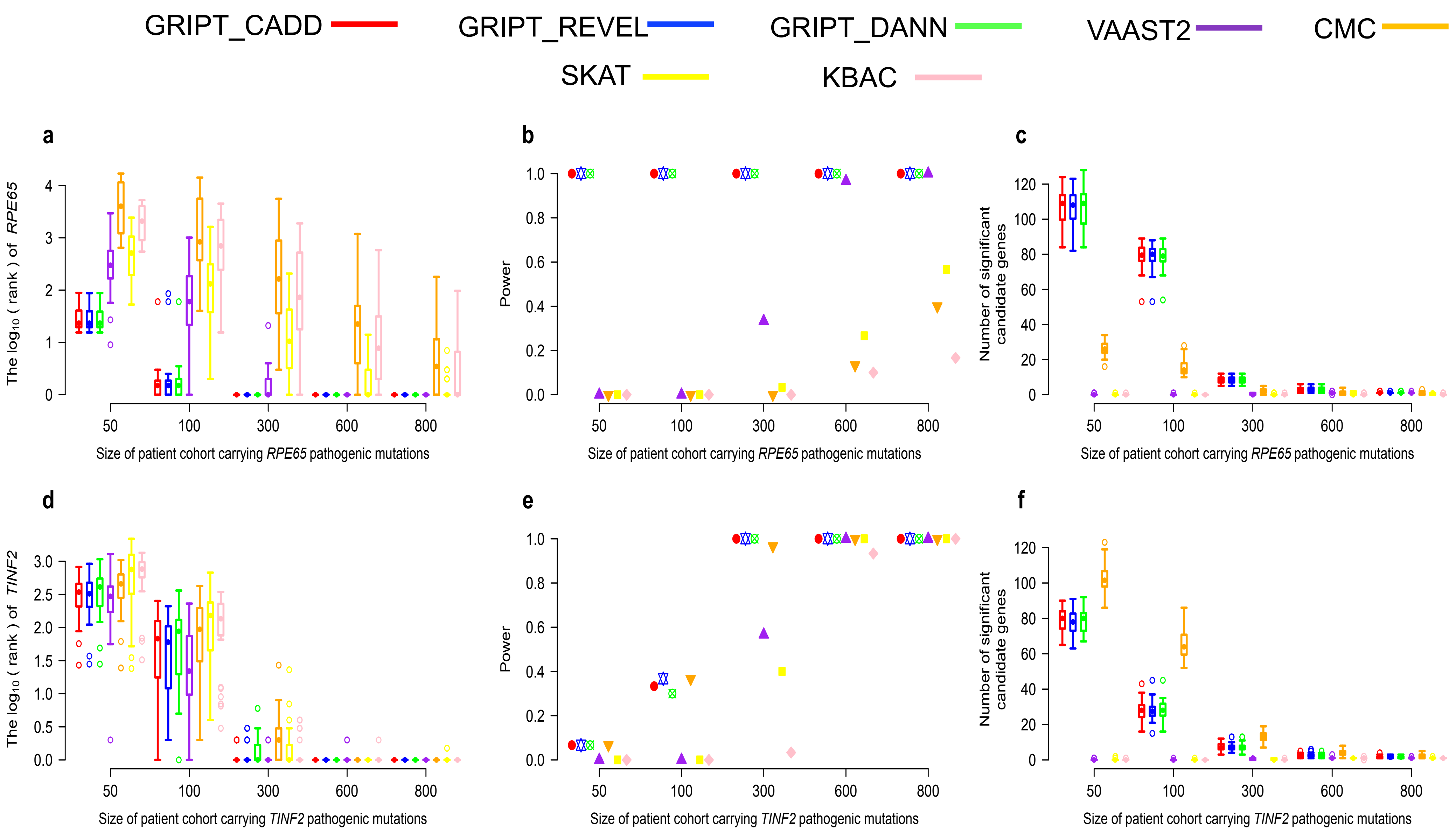

Figure S2. Test the impact of patient cohort sizes with REVEL and DANN scores

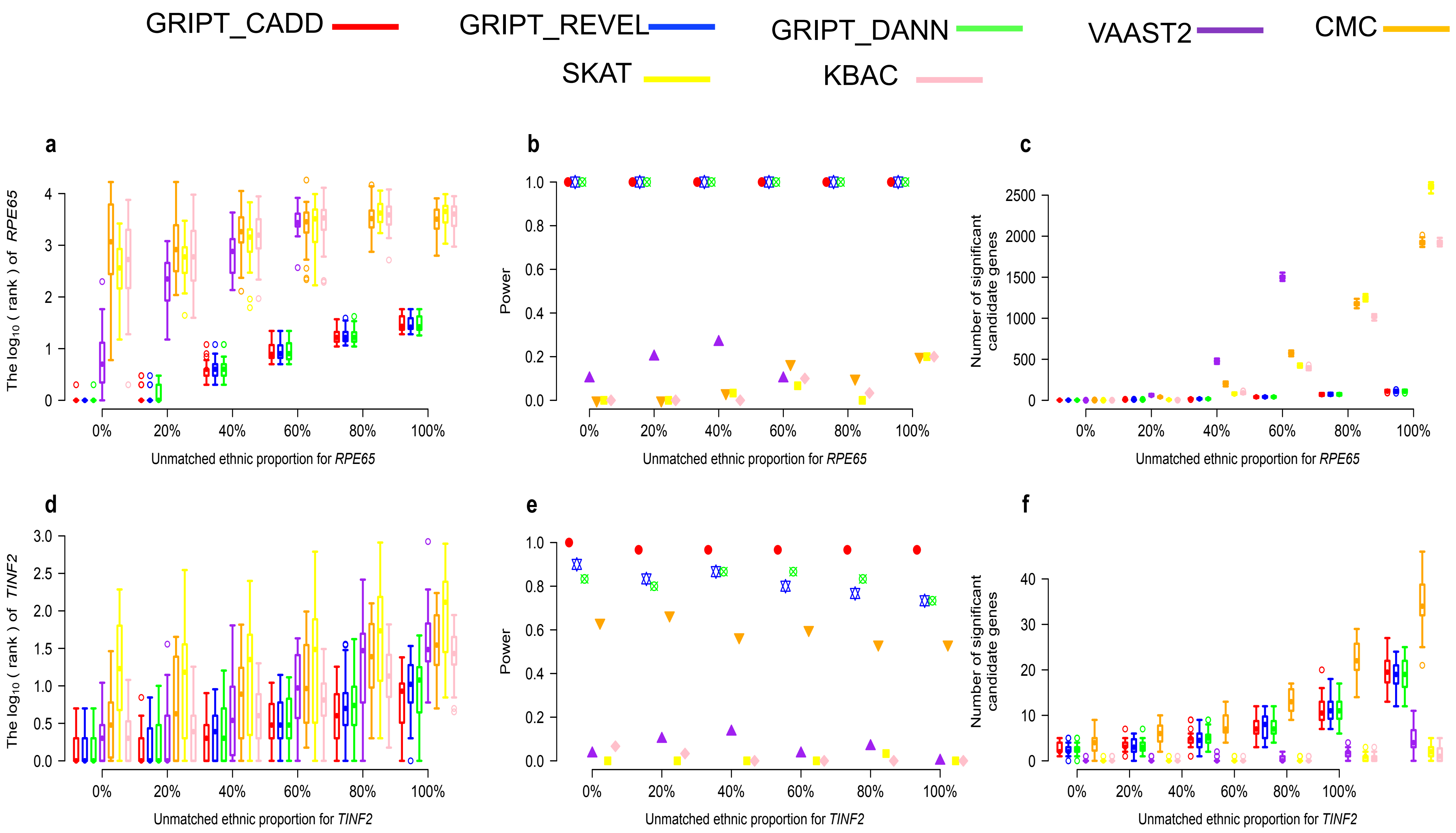

Figure S3. Test the impact of population stratification with REVEL and DANN scores

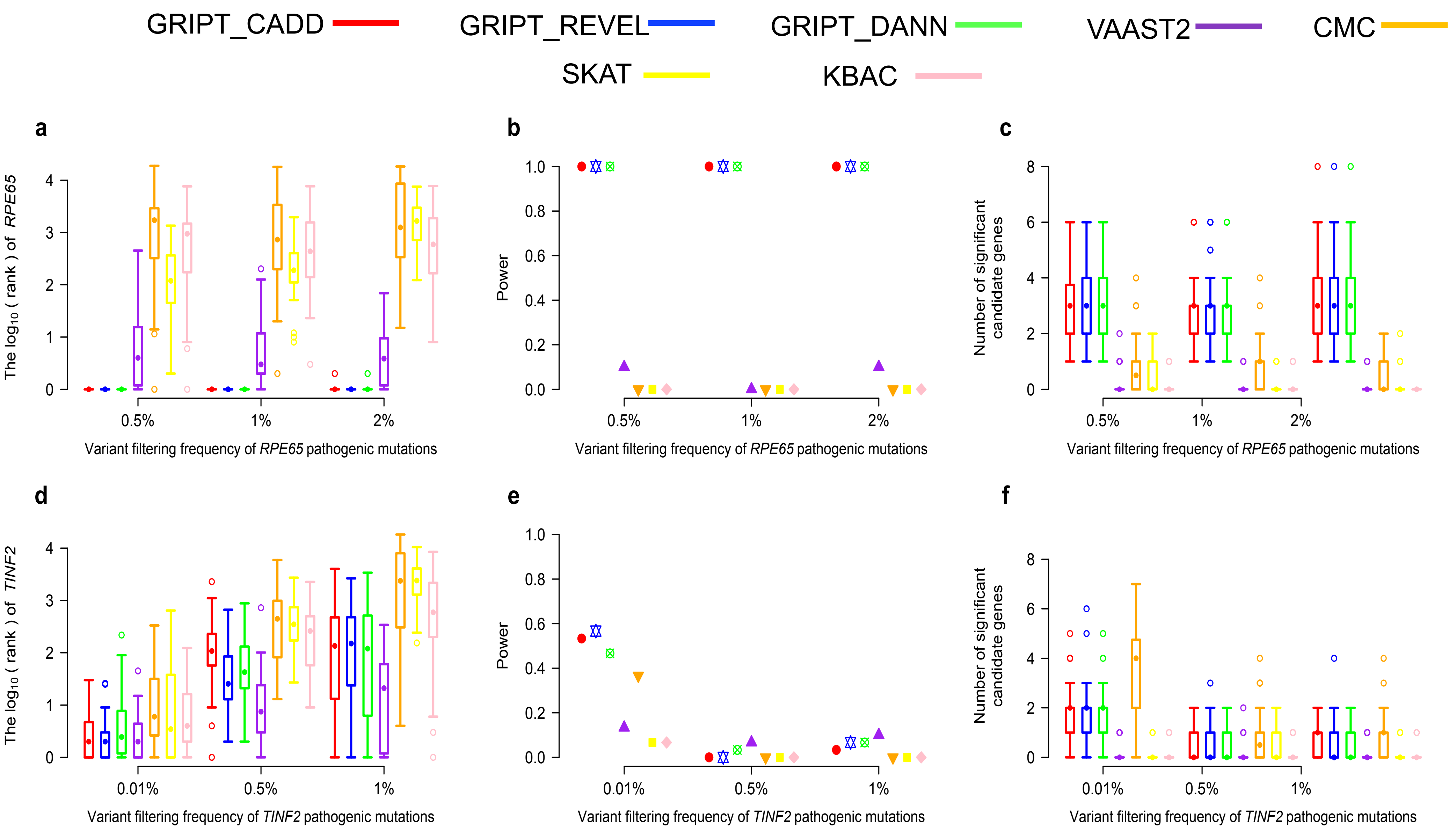

Figure S4. Test the impact of variant frequency filtering with REVEL and DANN scores

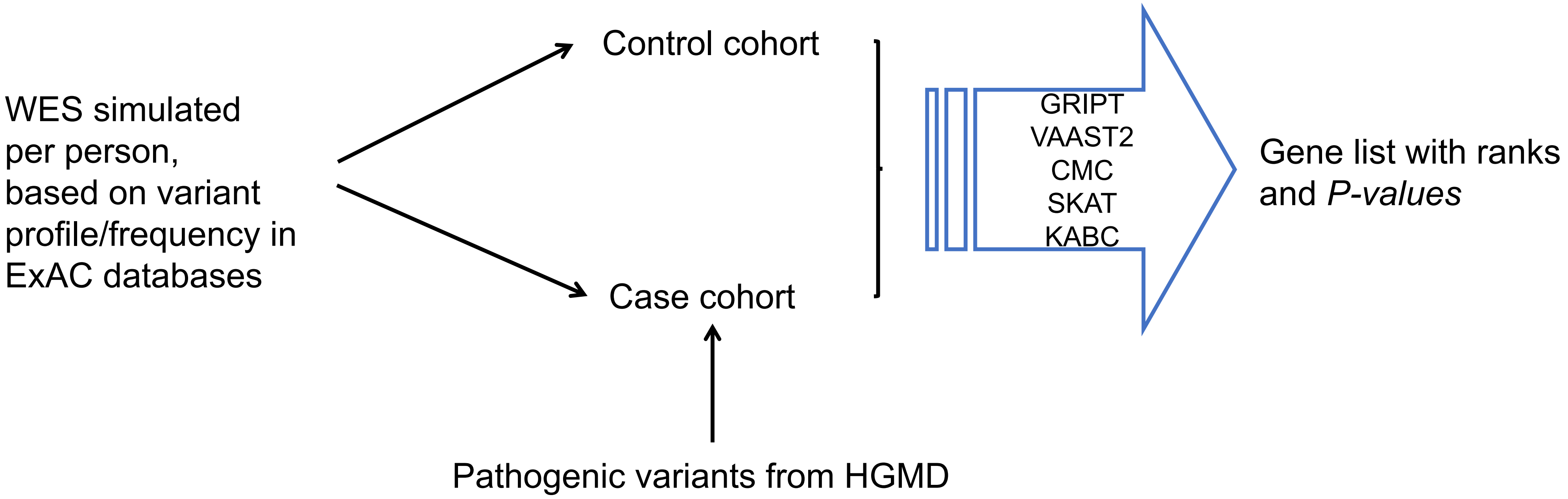

Figure S5. **The main procedure of simulation analysis**
